# Nitrogen Dioxide Inhalation Exposures Induce Cardiac Mitochondrial Reactive Oxygen Species Production, Impair Mitochondrial Function and Promote Coronary Endothelial Dysfunction

**DOI:** 10.3390/ijerph17155526

**Published:** 2020-07-30

**Authors:** Ahmed Karoui, Clément Crochemore, Najah Harouki, Cécile Corbière, David Preterre, Cathy Vendeville, Vincent Richard, Olivier Fardel, Valérie Lecureur, Jean-Marie Vaugeois, François Sichel, Paul Mulder, Christelle Monteil

**Affiliations:** 1 Univ Rouen Normandie, Univ Caen Normandie, Normandie Univ, ABTE UR4651, 76 000 Rouen, France; ahmed.karoui@inserm.fr (A.K.); clement.crochemore@pasteur.fr (C.C.); cecile.corbiere@univ-rouen.fr (C.C.); cathy.vandeville@univ-rouen.fr (C.V.); jean-marie.vaugeois@univ-rouen.fr (J.-M.V.); 2 Univ Rouen Normandie, Institut National de la Santé et de la Recherche Médicale, Normandie Univ, ENVI UMR1096, 76 000 Rouen, France; najah.harouki@hotmail.com (N.H.); vincent.richard@univ-rouen.fr (V.R.); paul.mulder@univ-rouen.fr (P.M.); 3 CERTAM, 1 rue Joseph Fourier, 76 800 Saint-Etienne du Rouvray, France; david.preterre@certam-rouen.com; 4 Univ Rennes, EHESP, Irset (Institut de recherche en santé, environnement et travail)—UMR_S 1085, CHU Rennes, Inserm, 35 000 Rennes, France; olivier.fardel@univ-rennes1.fr (O.F.); valerie.lecureur@univ-rennes1.fr (V.L.); 5 Pôle Biologie, Rennes University Hospital, 35 203 Rennes, France; 6 Univ Caen Normandie, Univ Rouen Normandie, Normandie Univ, ABTE UR4651, 14 000 Caen, France; francois.sichel@unicaen.fr; 7 Centre François Baclesse, 14 000 Caen, France

**Keywords:** air pollution, nitrogen dioxide, mitochondria, endothelial dysfunction, coronary arteries, ROS, cardiovascular

## Abstract

Traffic air pollution is a major health problem and is recognized as an important risk factor for cardiovascular (CV) diseases. In a previous experimental study, we showed that diesel exhaust (DE) exposures induced cardiac mitochondrial and CV dysfunctions associated with the gaseous phase. Here, we hypothesized that NO_2_ exposures to levels close to those found in DE induce a mitochondrial reactive oxygen species (ROS) production, which contribute to an endothelial dysfunction, an early indicator for numerous CV diseases. For this, we studied the effects of NO_2_ on ROS production and its impacts on the mitochondrial, coronary endothelial and cardiac functions, after acute (one single exposure) and repeated (three h/day, five days/week for three weeks) exposures in Wistar rats. Acute NO_2_ exposure induced an early but reversible mitochondrial ROS production. This event was isolated since neither mitochondrial function nor endothelial function were impaired, whereas cardiac function assessment showed a reversible left ventricular dysfunction. Conversely, after three weeks of exposure this alteration was accompanied by a cardiac mitochondrial dysfunction highlighted by an alteration of adenosine triphosphate (ATP) synthesis and oxidative phosphorylation and an increase in mitochondrial ROS production. Moreover, repeated NO_2_ exposures promoted endothelial dysfunction of the coronary arteries, as shown by reduced acetylcholine-induced vasodilatation, which was due, at least partially, to a superoxide-dependent decrease of nitric oxide (NO) bioavailability. This study shows that NO_2_ exposures impair cardiac mitochondrial function, which, in conjunction with coronary endothelial dysfunction, contributes to cardiac dysfunction. Together, these results clearly identify NO_2_ as a probable risk factor in ischemic heart diseases.

## 1. Introduction

Air pollution is a major health problem and is recognized as an important risk factor for cardiovascular (CV) diseases [1]. Among air pollutants, nitrogen dioxide (NO_2_) is a reactive gas and a primary pollutant originating from a variety of sources, especially from the combustion of fossil fuel and present in diesel exhaust (DE) [2]. This pollutant is regarded as a marker of motorized road traffic pollution and has been associated with CV adverse health outcomes [3]. However, close correlations between NO_2_ and other air pollutants, mainly particulate matter (PM), make it difficult to identify adverse effects due to NO_2_ alone [4]. Moreover, there is limited experimental evidence from controlled human exposure and animal toxicology studies for NO_2_, which have focused largely on respiratory parameters. It should also be stressed that NO_2_ air monitor networks are sparse or non-existent in many countries [5]. As a result, associations between NO_2_ exposures and CV health may be underestimated.

Some experimental investigations have shown the presence of biomarkers for CV effects, including markers for oxidative stress, inflammation, cell adhesion and endothelial dysfunction after NO_2_ exposure in rodents [6,7]. In term of vascular function, a controlled human exposure study did not find any impact on brachial artery reactivity after one h exposure to four ppm NO_2_ [8], whereas in a previous study, one hour exposure to diluted DE, including both gaseous and particulate phases, induced a vascular dysfunction and impaired endogenous fibrinolysis in men [9]. In the same way, Lucking et al. [10] demonstrated the preventive action of a particle trap on the vascular and prothrombotic effects of DE inhalation, suggesting the involvement of PM in these effects. Although these studies showed that the acute adverse vascular effects of air pollution are mediated by components other than NO_2_, other studies suggested that NO_2_ itself contributes to the deleterious effects of DE [11,12], but the CV functional consequences of these exposures remain to be established. 

In a previous study, we showed that DE-induced cardiac mitochondrial and CV dysfunctions were associated with the gaseous phase, since the observed effects were similar upstream or downstream of a particle trap [13]. However, the question arises as to whether NO_2_ may be involved in these CV effects. Mitochondrial defect is a prominent feature of most CV diseases, such as heart failure, cardiac hypertrophy, ischemic heart disease and atherosclerosis [14]. Dysfunctional mitochondria might generate excessive levels of reactive oxygen species (ROS) and one of the consequences is a decline in endothelial NO bioavailability [15,16]. In the area of air pollution, mitochondria are a potential target because they have not only key roles in cardiac cell functions, but they also produce ROS that may contribute to oxidative stress, a major determinant for air pollutant toxicity [17,18]. 

Based on these observations, it is tempting to speculate that NO_2_ exposures to levels close to those found in DE induce mitochondrial dysfunction, which contributes to the alteration of coronary microvascular reactivity associated with a cardiac dysfunction. To test this hypothesis, we studied the effects of NO_2_ inhalation in rats on: 1. cardiac mitochondrial function and ROS production, 2. cardiac function, 3. endothelium-dependent vasodilator responses of coronary arteries and 4. the role that ROS could play in these alterations. These experiments were performed after acute or repeated exposures, with the aim of establishing an exposure-response relationship and finally of showing the contribution of NO_2_ in air pollution-mediated cardiovascular effects.

## 2. Materials and Methods

*Animals and animal care*. This study conforms to the Guide for the Care and Use of Laboratory Animals published by the US National Institutes of Health (NIH Publication No. 85-23, revised 1996) as well as European legislation and was approved by the ethics committee CENOMEXA n°54 (authorization number n°01796.02). Male Wistar rats 9–11 weeks old were purchased from Janvier Labs (Le Genest Saint Isle, France) and maintained in the animal facility at 21 °C on a 12-h light/dark cycle with free access to water and food. 

**Figure 1:**
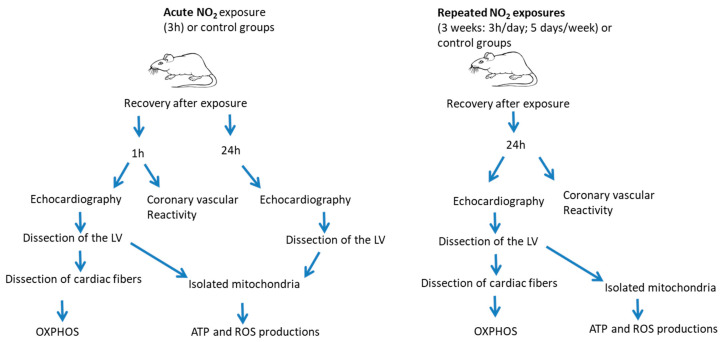
Experimental study design. LV: Left ventricle; OXPHOS: mitochondrial oxidative phosphorylation capacity; ROS: reactive oxygen species.

*Whole-body inhalation exposure.* Animals were randomly divided into six groups and placed in inhalation chambers during the exposures in the animal facility, as previously described [19]. Four groups were used for acute exposures to 5 ppm NO_2_ or to clean air for a single 3 h period and the evaluations were performed after a recovery period of 1 h or 24 h in clean air in the animal facility; two groups were used for repeated exposures to clean air or to 5 ppm NO_2_ 3 h/day, 5 days/week during 3 weeks and the evaluations were performed at 1 day post-exposure after a recovery period in clean air in the animal facility, which is vented with an high-efficiency particulate air and active charcoal filter to remove any pollutants from the outside. Clean air exposure was conducted using dry compressed air (dew point −70 °C) particle-free (<1 particle/cc using a condensation particle counter) with active charcoal treatment. Relative humidity (RH) is set to 50% RH with a steam humidification control system. Downward Mass Flow Controller is protected using cryotrap and adequate filtering. The experimental design is presented in Figure 1. To comply with the established “3R” (Reduce, Refine and Replace) principles, only the minimum number of animals has been used. At least *n* = 6 rats per group were used for the echocardiographic and biological assessments and at least *n* = 4 rats per group were used for the coronary vascular reactivity.

The atmosphere of NO_2_ in the inhalation chambers was obtained by mixing NO_2_ gas (Air Liquid Product, France) with the filtered ambient air in a dilution column upstream of the inhalation chambers, and continuously measured during the exposures by a chemiluminescence analyzer environmental range (AC31M, Environnement SA, Poissy, France). The calibration of the chemiluminescence analyzer was done using a known concentration of NO_2_. The experimental device to generate the NO_2_ atmosphere was placed in a room outside of the animal facility in order to avoid transfer of potentially stressful noise.

The NO_2_ concentration is in the range of the concentrations measured in our previous studies conducted with diluted DE emitted under dynamic conditions (“New European Driving Cycle” NEDC) and characterized by an average level of 3.3 ppm NO_2_ [13,20]. The choice of 5 ppm NO_2_ was also based on previous experimental studies demonstrating effects on cardiac biochemical parameters [6].

*Cardiac function evaluations.* Echocardiographic assessments were conducted blind to the animal group and performed in sedated rats (100–135 mg/kg ketamine; 3 mg/kg xylazine) after different recovery periods. Left ventricular (LV) dimensions, and function were assessed with a Vivid 7 ultrasound device (General Electric Healthcare, France), as previously described [21]. Briefly, cardiac ventricular dimensions were measured using M-mode tracings recorded from a two-dimensional short-axis view at the level of the papillary muscles. Echocardiography provided measurements of left ventricular (LV) end-diastolic (LVedd) and end-systolic (LVesd) diameters and posterior wall thickness at diastole (PWEDT) and at systole (PWEST). LV systolic function was assessed by the fractional shortening (FS) [(LVedd-LVesd)/LVedd] x 100. Velocity-time integral was measured by pulsed-wave Doppler, and cardiac output (CO) was calculated as CO = aortic velocity-time integral × [(π × LV outflow diameter)2/4]/100 × heart rate.

*Vascular function evaluation*. Coronary vascular reactivity was evaluated by myograph (Dual Wire Myograph System; Danish Myo Technology), as previously described [22]. In brief, the heart was placed in cold, oxygenated Krebs buffer. A segment of the septal coronary artery, 1 mm long and 100 μm in diameter, was carefully dissected and mounted in a small vessel myograph for isometric tension recording. All measurements were performed after vessel contraction with 10^−5^ M serotonin, and pharmacological inhibitors were applied for 30 min before assessing the relaxant responses. The endothelium-dependent relaxations to acetylcholine (10^−9^ to 10^−4.5^ M) were assessed in the absence and in the presence of the NOS inhibitor Nω-nitro-l-arginine (L-NNA; 10^−4^ M) or superoxide dismutase (SOD; 200 UI/mL). Endothelium-independent relaxations to the NO donor sodium nitroprusside (SNP; 10^−9^ to 10^−4.5^ M) were assessed.

*Cardiac mitochondrial evaluations*. For the mitochondrial evaluations, two types of sample were freshly prepared from LV as previously described [13,23]: permeabilized cardiac fibers for evaluations of mitochondrial oxidative phosphorylation capacity (OXPHOS) and isolated mitochondria for ATP and ROS measurements. The OXPHOS capacity was evaluated in situ on permeabilized cardiac fibers, in order to maintain the mitochondria in their normal intracellular position and their interactions with other organelles. OXPHOS was measured polarographically at 22 °C using a Clark-type oxygen electrode (Strathkelvin Instruments, Scotland, UK). Briefly, permeabilized cardiac fibers were added under continuous stirring in an oxygraphic cell containing R-buffer (2.77 mM CaK_2_EGTA, 7.23 mM K_2_EGTA (100 nM free Ca^2+^), 1.38 mM MgCl_2_ (1 mM free Mg^2+^), 20 mM taurine, 0.5 mM dithiotreitol, 90 mM potassium-methane sulfonate, and 20 mM imidazole, 10 mM sodium-methane sulfonate and 2 mg/mL bovine serumalbumine, pH 7.1) and malate/glutamate (4 mM/10 mM). O_2_ consumption was measured in the absence (state 2) and the presence of 2 mM adenosine diphosphate (ADP) (state 3 with complex I-linked substrates). Then, 2 mM amytal and 10 mM succinate were added (state 3 with complex II-linked substrates). O_2_ consumption rates are given in micromoles of O_2_ per minute per mg of proteins. The acceptor control ratio (ACR), an index of oxidation-phosphorylation coupling, was also calculated as the ratio of state 3 to state 2.

For cardiac mitochondrial ATP production, freshly isolated subsarcolemmal (SSM) and interfibrillar (IFM) mitochondria were incubated at 25 °C in R-tampon with 10 mM glutamate and 4 mM malate for 10 min under continuous stirring to eliminate sample residual ADP. Then, 2 mM ADP was added and samples were collected as previously described [13]. The rate of ATP production was evaluated by a bioluminescence assay kit (Roche Diagnostics, France) according to the manufacturer’s protocol. Values were normalized to the protein concentration of each sample.

Superoxide production was evaluated from SSM or IFM preparations by electron paramagnetic resonance (EPR) spectroscopy using the spin probes: 1-hydroxy-3-methoxycarbonyl-2,2,5,5-tetramethylpyrrolidine (CMH, Noxygen, Germany) as previously described [23,24]. Briefly, IFM and SSM were incubated at 37 °C for 1 h in Krebs–Hepes buffer (0.1 M NaCl, 5 mM KCl, 2.5 mM CaCl_2_, 1.2 mM MgSO_4_, 25 mM NaHCO_3_, 1 mM KH_2_PO_4_, 5.6 mM D-(+)-glucose, 20 mM Na–Hepes, pH 7.4) containing 25 μM deferoxamine, 5 μM diethyldithiocarbamate, and supplemented with 0.5 mM CMH, 10 mM Glutamate, 4 mM malate and 2 mM ADP. The oxidation of CMH into the stable 3-methoxycarbonylproxyl (CM°) was recorded using a MiniScope MS-200 X-band spectrometer (Magnettech, Germany). The EPR instrumental settings for field scan were as follows: Bo-field 3356.98 G, microwave power 1 mW, microwave attenuation 20 dB, modulation frequency 9.74 GHz, modulation amplitude 5 G, scan time 60 s. Intensity of the spectra was measured from the height of the central line. EPR data are expressed as arbitrary units/mg mitochondrial proteins.

### Statistical Analysis

All values are expressed as means ± standard error of mean (SEM). Student’s *t*-test was performed to compare means between control and NO_2_ exposed groups. Statistical analyses were performed using GraphPad Prism (version 7.04, GraphPad software, USA). Differences were considered statistically significant when *p* < 0.05.

## 3. Results

### 3.1. Acute NO_2_ Exposure Induced a Rapid but Reversible Cardiac Response

**Figure 2:**
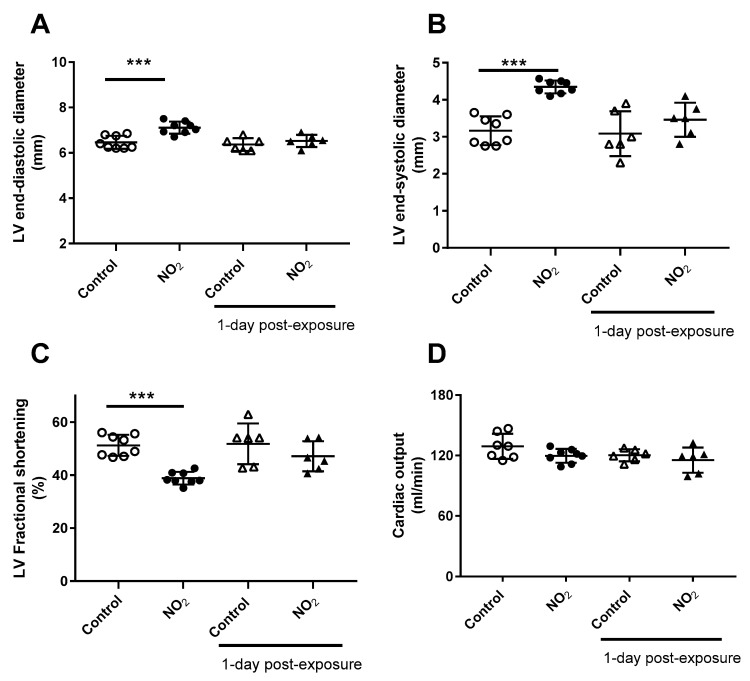
Echocardiographic parameters and endothelial function after acute NO_2_ exposure. The diagram shows echocardiographic measurements of left ventricle (LV) end-diastolic diameter (**A**), LV end-systolic diameter (**B**), LV fractional shortening (**C**) and cardiac output (**D**), measured after acute NO_2_ exposure or 1-day post-exposure when specified. *** *p* < 0.001 between corresponding group.

LV function was evaluated by echocardiography after acute exposure to NO_2_ (Figure 2A–D). Relative to control group, single exposure to NO_2_ induced a significant increase in LV diastolic and systolic diameters (10 and 38%, respectively), and a decrease in fractional shortening (−23%), after 1 h of recovery period. This effect appeared transient since a 24 h recovery period post-acute exposure erased the increase in LV diastolic and systolic diameters and the decrease in fractional shortening were no longer observed. Cardiac output (Figure 2D) remained unchanged after NO_2_ exposure, whatever the recovery period.

**Figure 3:**
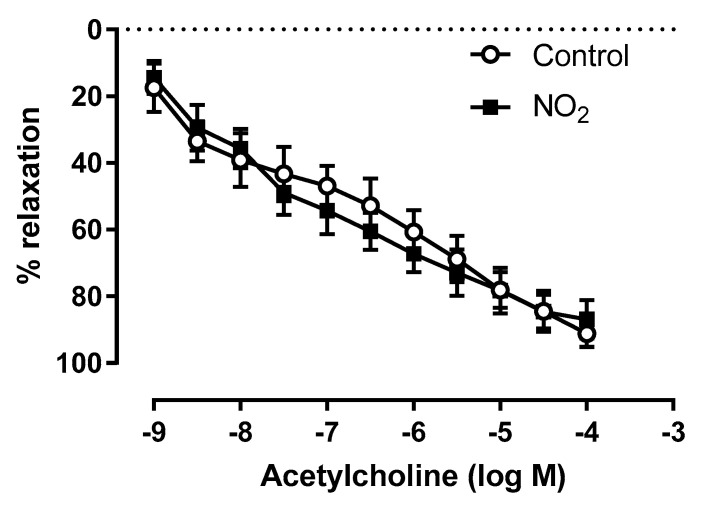
Coronary vasodilation measured after acute NO_2_ exposure.

To gain further insight into the putative impact of NO_2_ exposures on vascular function, we next investigated coronary endothelial function. Coronary arteries were isolated from the hearts of control and NO_2_-exposed rats for vascular reactivity assessment, as a measure of endothelium-dependent coronary vasodilatation after serotonin pre-constriction and the addition of acetylcholine. Acute NO_2_ exposure did not impair endothelial function in coronary arteries, as shown by comparable endothelial relaxation in both control and acute NO_2_ exposure groups (Figure 3).

### 3.2. Acute NO_2_ Exposure Induced Rapid but Transitory Cardiac Mitochondrial ROS Production without Mitochondrial Dysfunction

**Figure 4:**
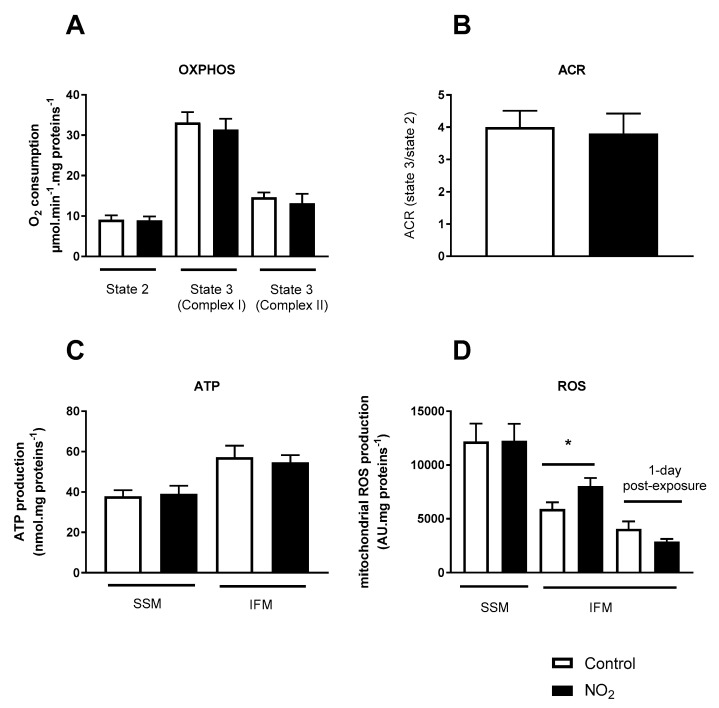
Cardiac mitochondrial function and ROS production after acute NO_2_ exposure. (**A**) Mitochondrial OXPHOS evaluated by O_2_ oxygen consumption assessment in cardiac permeabilized fibers. State 2 respiration with complex I-linked substrates (Glutamate/Malate); State 3 respiration with complex I-linked substrates (Glutamate/Malate); State 3 respiration with complex II-linked substrates (Succinate); (**B**) Acceptor control ratio (ACR) with complex I-linked substrates (**C**) adenosine triphosphate (ATP; nmol/mg proteins) and (**D**) reactive oxygen species (ROS) productions (A.U/mg proteins) were measured from freshly isolated subsarcolemmal (SSM) and interfibrillar (IFM), 1 h or 1-day post-exposure, when specified, after acute NO_2_ or air (control) exposure. * *p* < 0.05.

To investigate mitochondrial function after NO_2_ exposure, the OXPHOS capacity was investigated in situ from permeabilized cardiac fibers in order to maintain the mitochondria in their normal intracellular position and their interactions with other organelles. Acute NO_2_ exposure did not affect the mitochondrial function, since ADP-independent respiration with glutamate and malate (state 2) was similar between the groups, as well as ADP-dependent respiration with glutamate and malate (state 3 complex I) or succinate (state 3 complex II) as substrates (Figure 4A). In these conditions, oxidation-phosphorylation coupling, determined by the ratio of respiration rate before and after the addition of ADP (acceptor control ratio ACR, state 3 (complex I)/state 2), remained unchanged (Figure 4B).

To further explore the effect of acute NO_2_ exposure on mitochondrial function, we next measured both ATP production rates and superoxide production in cardiac mitochondrial subpopulations, subsarcolemmal (SSM) and interfibrillar (IFM) mitochondria. Acute NO_2_ exposure did not alter ATP production rates, confirming the absence of a mitochondrial dysfunction (Figure 4C), although a temporary increase in mitochondrial ROS production was observed specifically in IFM. Indeed, ROS levels were similar between the groups, 1-day post-exposure (Figure 4D).

### 3.3. Repeated NO_2_ Exposures Impaired Cardiovascular Responses

**Figure 5:**
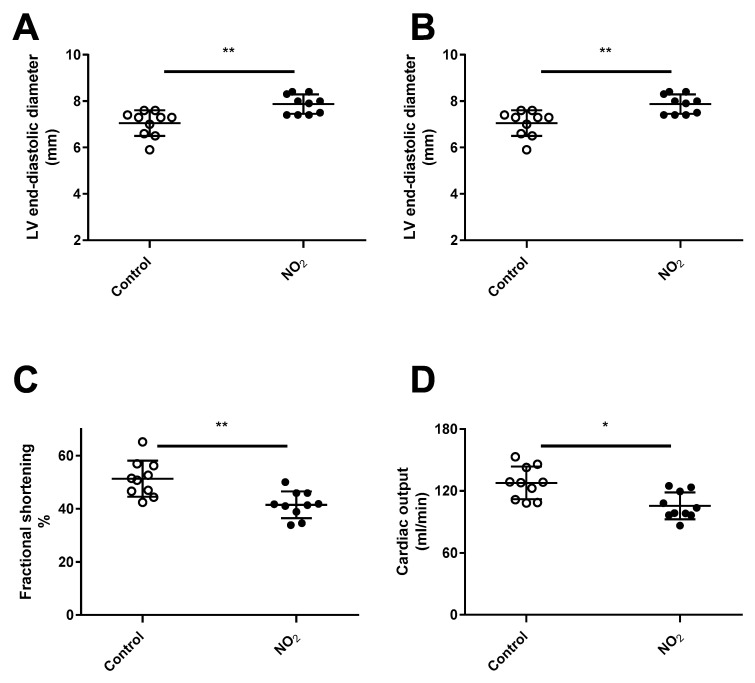
Echocardiographic parameters after repeated NO_2_ exposures. Echocardiographic assessments were performed at 1-day post-exposure after 3 weeks (3 h/day, 5 days/week) of NO_2_ exposures. The diagram shows echocardiographic measurements of left ventricle (LV) end-diastolic diameter (**A**), LV end-systolic diameter (**B**), LV fractional shortening (**C**) and cardiac output (**D**). * *p* < 0.05, ** *p* < 0.01, *** *p* < 0.001, vs. control.

We next wanted to determine whether the duration of NO_2_ exposure has differential effects on cardiovascular function. Three weeks of NO_2_-exposures caused a sustained cardiac effect, since 24 h after the 3-week exposure, LV diastolic and systolic diameters (12% and 34%, respectively) as well as LV fractional shortening and cardiac output (−20% and −17%, respectively) were severely altered compared to time-matched controls (Figure 5).

**Figure 6:**
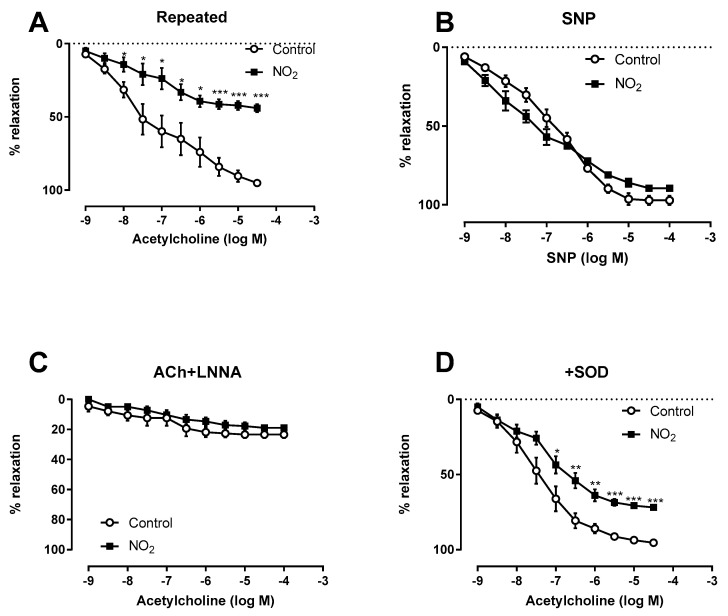
Coronary function after repeated NO_2_ exposures. Coronary function was assessed at 1-day post-exposure after 3 weeks (3 h/day, 5 days/week) of NO_2_ exposures. Vasorelaxation induced by acetylcholine (**A**) or sodium nitroprusside (SNP) (**B**), or acetylcholine after preincubation with either NG-nitro-L-arginine (Ach + L-NNA) (**C**) or superoxide dismutase (+SOD) (**D**) of isolated coronary arteries from control (open circles) or NO_2_-exposed rat (solid squares). * *p* < 0.05, *** *p* < 0.001 vs. control.

In parallel with this cardiac dysfunction, repeated NO_2_ exposures impaired endothelium-dependent relaxation, illustrated by the decrease of coronary relaxation in the repeated NO_2_ exposure group (Figure 6A). No differences were observed between groups upon sodium nitroprusside (SNP)-induced relaxation (Figure 6B).

Incubation of coronary arteries with the NOS inhibitor L-NNA markedly reduced the relaxing response in both groups (Figure 6C). Incubation of coronary arteries with superoxide dismutase did not modify acetylcholine responses in the control group but improved the impaired relaxation observed after repeated NO_2_ exposure (Figure 6D).

### 3.4. Repeated NO_2_ Exposures Impaired Mitochondrial Function and Mitochondrial ROS Production

**Figure 7:**
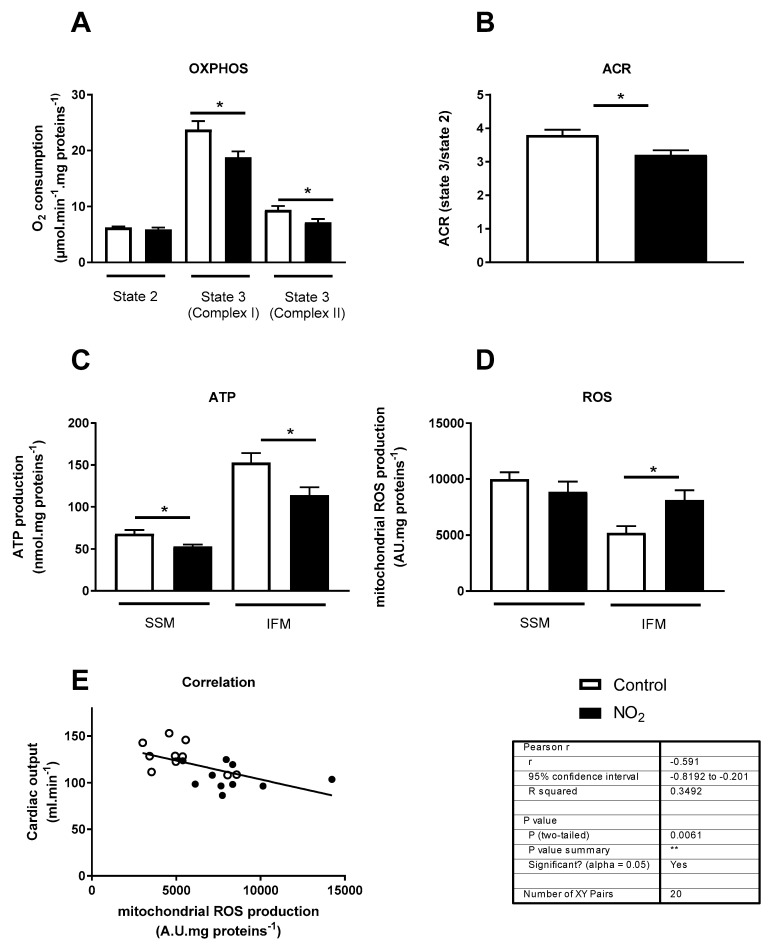
Cardiac mitochondrial function, ROS production and correlation with cardiac function, at 1-day post-exposure after repeated NO_2_ exposures. (**A**) Mitochondrial OXPHOS evaluated by O_2_ oxygen consumption assessment in permeabilized fibers. State 2 respiration with complex I-linked substrates (Glutamate/Malate); State 3 respiration with ADP and complex I-linked substrates (Glutamate/Malate); State 3 respiration with ADP and complex II-linked substrates (Succinate); (**B**) ACR with complex I-linked substrates (**C**) ATP (nmol/mg proteins) and (**D**) ROS productions (A.U/mg proteins) were measured from freshly isolated SSM and IFM. (**E**) Correlation between cardiac output and interfibrillar mitochondrial ROS production. * *p* < 0.05.

In order to determine whether repeated NO_2_ exposure affected mitochondrial function, we performed exposures during three weeks and the assessments were performed at 1-day post-exposure. Figure 7 displays cardiac mitochondrial OXPHOS capacity after repeated NO_2_ exposures.

Respiration with either complex I or complex II substrates was reduced by 21 and 23%, respectively (Figure 7A) after repeated NO_2_ exposure. In line with these results of state 3 respiration, ACR was lower in NO_2_- compared to air-exposed rats (Figure 7B). The ATP production rate decreases observed in both SSM and IFM fractions confirmed this mitochondrial dysfunction (Figure 7C). An increase in mitochondrial superoxide production was observed specifically in IFM (Figure 7D) and was correlated with the decrease in cardiac output (Figure 7E).

## 4. Discussion

The results of this study show that acute NO_2_ exposure induced an early, but transitory mitochondrial superoxide production associated with reversible impairment of cardiac function, whereas repeated exposures induced mitochondrial and cardiac dysfunctions, which persisted for 24 h after the last exposure. Moreover, repeated NO_2_ exposures impaired ACh-mediated dilatation in coronary arteries, an effect that was due to a decrease in NO bioavailability caused, at least partially, by increased superoxide production. Taken together, these data provide the first evidence that NO_2_ exposures impaired cardiac mitochondrial function, which, in conjunction with coronary endothelial dysfunction, contributes to a sustainable cardiac dysfunction.

Air pollution has long been recognized as a major CV risk, due particularly to fine particulate matter (PM_2.5_) derived from combustion sources, and road traffic and diesel exhaust (DE). However, the potential effects of a co-pollutant gases, such as NO_2_, has been neglected. In a previous study, we demonstrated that removing particles from the DE aerosol did not protect against DE-induced cardiac and mitochondrial dysfunctions, revealing an important implication of the gaseous phase in this response [13]. In this present study, acute NO_2_ exposure at a concentration close to that measured in DE induced a cardiac impairment characterized by an LV dilatation and associated with reduced LV fractional shortening, reflecting a possible loss of contractility. This LV dysfunction appeared only moderate since cardiac output was not modified. Moreover, this effect was reversible since, after 1 day post-exposure, echocardiographic parameters were comparable to those measured in control group. However, cardiac dysfunction persisted and worsened after repeated exposure to NO_2_ over three weeks. These evaluations were made at day-1 post-exposure, excluding the deleterious direct effect of NO_2_ as observed 1 h after acute NO_2_ exposure. These results are consistent with a cohort study that has shown an association between past exposure to NO_2_ and PM_2.5_ and cardiac ventricular dilatation [25], a marker of adverse remodeling that often precedes heart failure development, stressing the irreversible effects of these exposures on the heart.

To identify the underlying cellular mechanisms involved in the cardiac dysfunction following NO_2_ exposure, we first focused on cardiac mitochondrial ROS and ATP production. Two spatially distinct mitochondria subpopulations have been observed in myocytes and may be associated with a specific response to pathological stimuli, indicating the role of IFM in contractile function [26,27]. The present study revealed a primary but reversible interfibrillar mitochondria-specific increase in ROS after acute exposure. This increase was not accompanied by a mitochondrial alteration, as evidenced by the maintenance of ATP synthesis capacity. The increase in mitochondrial ROS can reflect an adaptive event involved in cardio-protection, as previously demonstrated [28,29]. For example, Antonucci et al. [29] investigated the effects of mitochondrial ROS production using a mitochondria-targeted redox cycler MitoParaquat (MitoPQ) and showed that low levels of ROS are cardioprotective, while higher doses of MitoPQ resulted in a progressive alteration of mitochondrial function in vitro. The magnitude but also the repetition of the mitochondrial ROS production may result in the alteration of mitochondrial function and in worsening of cardiac function, as observed after repeated NO_2_ exposures. The strong correlation between mitochondrial ROS production and cardiac output associated with the appearance of mitochondrial dysfunction support this hypothesis. The fact that mitochondrial ROS production precedes the mitochondrial dysfunction suggest that this production is an early trigger event in the onset of mitochondrial defect. Indeed, we showed that respiration rate was affected in the hearts of rats exposed to repeated NO_2_ exposures. This is consistent with our previous study showing that repeated DE exposures induced an impairment of mitochondrial function associated with deficient cardiac contractility [13]. With regard to other combustion-related gas, CO [30] and SO_2_ [31] exposures also induced cardiac mitochondrial effects. Overall, these results suggest that mitochondria impairment contributes to the CV events linked to air pollution.

Then, we focused on coronary endothelial dysfunction. Indeed, despite the number of studies showing that air pollution, and more specifically DE particles, causes endothelial dysfunction [32,33,34], the contribution of the gaseous phase in these effects is underestimated and the effect of NO_2_ itself on endothelial function is unknown. After acute exposure, we did not observe any effect on endothelial function. This is in line with a previous study showing no impact on brachial artery reactivity after 1 h exposure of humans to 4 ppm NO_2_ [8]. Nevertheless, repeated NO_2_ exposures impaired relaxation to acetylcholine in coronary arteries from healthy rats. Endothelial NO participates in the control of vascular tone and changes observed in the reactivity of coronary arteries after NO_2_ exposures might be explained by a decrease in NO availability. As repeated NO_2_ did not modify relaxation induced by sodium nitroprusside, a NO donor, a direct effect of NO_2_ on vascular smooth muscle cells is unlikely. These results demonstrate that NO_2_ exposures impaired coronary endothelial cell function, an effect that was due to a reduction of endothelial NO availability. One possible mechanism of reduced NO availability might be explained by an increased superoxide production in arteries after NO_2_ exposures, since superoxide scavenging by SOD restored attenuated ACh-induced relaxation of coronary arteries. The reduction in NO-bioavailability and the resulting altered coronary function probably contributes to a decrease in myocardial perfusion, which is likely to contribute to hypoxia-induced ROS production and, as a probable consequence, causes the vicious circle of ROS-induced ROS release [35,36]. Although we did not evaluate the pro-inflammatory markers in the present study, we cannot exclude a contribution of circulating factors in these effects. A previous study suggested vascular toxicity mediated by pro-inflammatory circulating factors following exposure to NO_2_ [12]. These authors showed that plasma from humans exposed to 0.5 ppm NO_2_ was able to activate expression of cell adhesion molecules by coronary endothelial cells in culture [12]. Further studies are needed to determine the precise underlying mechanism.

In conclusion, we demonstrate for the first time that repeated NO_2_ exposures altered cardiac mitochondrial and cardiac function, as well as coronary vascular reactivity, because of endothelial dysfunction. Moreover, these results demonstrated that acute exposure to NO_2_ induced a mitochondrial ROS production, which could represent an early trigger event in the onset of cardiovascular defects after chronic exposures.

## Submission Declaration

The authors declare that the work described has not been published previously.
