# Correction: Karoui et al. Nitrogen Dioxide Inhalation Exposures Induce Cardiac Mitochondrial Reactive Oxygen Species Production, Impair Mitochondrial Function and Promote Coronary Endothelial Dysfunction. *Int. J. Environ. Res. Public Health* 2020, *17*, 5526

**DOI:** 10.3390/ijerph22040635

**Published:** 2025-04-18

**Authors:** Ahmed Karoui, Clément Crochemore, Najah Harouki, Cécile Corbière, David Preterre, Cathy Vendeville, Vincent Richard, Olivier Fardel, Valérie Lecureur, Jean-Marie Vaugeois, François Sichel, Paul Mulder, Christelle Monteil

**Affiliations:** 1 Univ Rouen Normandie, Univ Caen Normandie, Normandie Univ, ABTE UR4651, 76 000 Rouen, France; ahmed.karoui@inserm.fr (A.K.); clement.crochemore@pasteur.fr (C.C.); cecile.corbiere@univ-rouen.fr (C.C.); cathy.vandeville@univ-rouen.fr (C.V.); jean-marie.vaugeois@univ-rouen.fr (J.-M.V.); 2 Univ Rouen Normandie, Institut National de la Santé et de la Recherche Médicale, Normandie Univ, ENVI UMR1096, 76 000 Rouen, France; najah.harouki@hotmail.com (N.H.); vincent.richard@univ-rouen.fr (V.R.); paul.mulder@univ-rouen.fr (P.M.); 3 CERTAM, 1 rue Joseph Fourier, 76 800 Saint-Etienne du Rouvray, France; david.preterre@certam-rouen.com; 4 Univ Rennes, EHESP, Irset (Institut de recherche en santé, environnement et travail)—UMR_S 1085, CHU Rennes, Inserm, 35 000 Rennes, France; olivier.fardel@univ-rennes1.fr (O.F.); valerie.lecureur@univ-rennes1.fr (V.L.); 5 Pôle Biologie, Rennes University Hospital, 35 203 Rennes, France; 6 Univ Caen Normandie, Univ Rouen Normandie, Normandie Univ, ABTE UR4651, 14 000 Caen, France; francois.sichel@unicaen.fr; 7 Centre François Baclesse, 14 000 Caen, France

In the original publication [1], there was a mistake in Figure 5B as published. Figure 5B was a copy of Figure 5A. The corrected Figure 5B appears below.

A correction has been made to the first parentheses in the first paragraph of Section 3.3. Repeated NO_2_ Exposures Impaired Cardiovascular Responses: “(12% and 34%, respectively)”.

In the published publication, the affiliations for authors are no longer in line with current recommendations. The affiliations have therefore been updated. In addition to affiliations 1–6, the updated affiliations should include: Univ Caen Normandie, Univ Rouen Normandie, Normandie Univ, ABTE UR4651, 14 000 Caen, France. 

The authors state that the scientific conclusions are unaffected. This correction was approved by the Academic Editor. The original publication has also been updated.

## Figures and Tables

**Figure 5:**
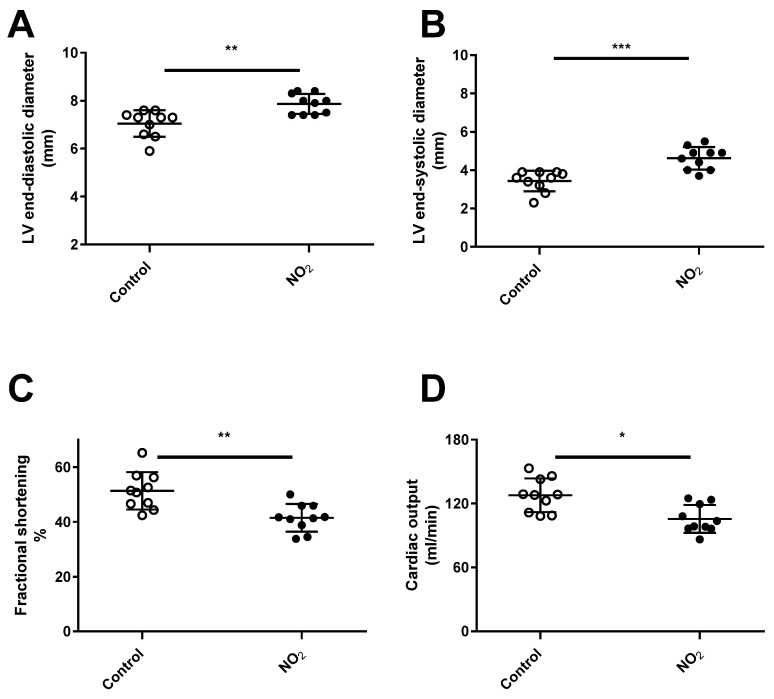
Echocardiographic parameters after repeated NO_2_ exposures. Echocardiographic assessments were performed at 1-day post-exposure after 3 weeks (3 h/day, 5 days/week) of NO_2_ exposures. The diagram shows echocardiographic measurements of left ventricle (LV) end-diastolic diameter (**A**), LV end-systolic diameter (**B**), LV fractional shortening (**C**) and cardiac output (**D**). * *p* < 0.05, ** *p* < 0.01, *** *p* < 0.001, vs. control.
